# Unusual side effect of cannabis use: acute abdomen due to duodenal perforation

**DOI:** 10.1186/s12245-016-0114-7

**Published:** 2016-07-07

**Authors:** Sevgi Buyukbese Sarsu

**Affiliations:** Department of Pediatric Surgery, Gaziantep Cengiz Gokcek Obstetrics and Children’s Hospital, 27560 Sehitkamil, Gaziantep Turkey

## Abstract

**Background:**

The chronic use of synthetic cannabinoids (SCs) which has become an increasingly prevalent problem can rarely cause gastric and duodenal ulcer because of their effects on gastric secretion and emptying.

Since peptic ulcer disease (PUD) is a rarely seen entity in patients who consult to the emergency service with complaints of abdominal pain, most of the physicians do not suspect of this clinical diagnosis. Perforation is a mortal complication of PUD, and early diagnosis and emergency surgery are life-saving procedures.

**Case presentation:**

A 16-year-old male patient was referred to our emergency service from another center with abdominal distension, complaints of abdominal pain, and bilious vomiting. His medical history revealed that he had been regularly using bonsai for the past 3 years. Plain abdominal radiograms of standing position revealed subdiaphragmatic free air, then we performed laparotomy which disclosed perforation of the first part of the duodenum. Surgical intervention with omental patch and primary closure (Graham patch) was successful. The patient who underwent nasogastric decompression and received antibiotherapy had not experienced any complication during the postoperative follow-up period.

**Conclusion:**

Herein, as an unusual manifestation, a patient who developed duodenal perforation following chronic SC use has been reported.

In adolescent patients admitted with PUD or its complications to the emergency services, it is important to inquire for the use of addictive substances which are increasingly prevalent in order to determine the etiology.

## Background

Cannabinoids (also called) cannabis are plant-derived addictive substances which have been used widely for thousands of years [1]. Despite their known therapeutic effects, because of their addictive properties, they cause the most prevalent health problem in the world.

The widespread use of cannabinoids or their synthetic derivatives exert many adverse effects on the health of human beings including pulmonary, endocrine, and cardiovascular pathologies, as well as cognitive and behavioral disorders. Synthetic cannabinoids (SCs) first emerged in the year 2004, and in a short time, it has become popular especially among adolescents [2]. In Turkey, it is known as “bonsai” [3].

Their widespread effects on the gastrointestinal system manifest themselves through their specific receptors in the brain and bowels. As a result of their chronic use, delay in gastric emptying and hyperemesis can be enumerated among their other harmful gastric effects. In the literature, development of acute pancreatitis following cannabinoid use has been reported [4]. In patients with acute pancreatitis, increased probability of developing peptic ulcer disease (PUD) has been found. In this case report, we indicated unusual clinical condition of an adolescent who was admitted to the emergency service with symptoms of acute abdomen.

## Case report

A 16-year-old male patient was referred to our emergency service from another center with abdominal distension, complaints of gradually increasing abdominal pain, and bilious vomiting persisting occasionally for the previous 15 days. The patient came from Southeastern Anatolia. His medical history revealed that he had been regularly using bonsai for the previous 3 years. His family was not aware of his addiction. On physical examination, abdominal distension, widespread tenderness, abdominal guarding, and washboard abdomen were detected on palpation. His biochemical parameters at his admission into the hospital were as follows: white blood cell count, 8.42 × 10^9^/μL (neutrophils, 64.6 %; lymphocytes, 29.4 %); CRP, 0.23 mg/L; hemoglobin, 10.3 g/dL; Htc, 33.2 %; platelet count, 589 × 10^9^/μL; glucose, 114 mg/dL; Na, 135 mEq/L; K, 4.17 mmol/L; Cl 100 mmol/L; aspartate transaminase, 18 U/L; alanine transaminase, 7 U/L; lactate dehydrogenase, 261 U/L; BUN, 10.3 mg/dL; and creatinine, 0.81 mg/dL. History of trauma, alcohol consumption, regular drug use, and chronic disease could not be elicited.

**Fig. 1:**
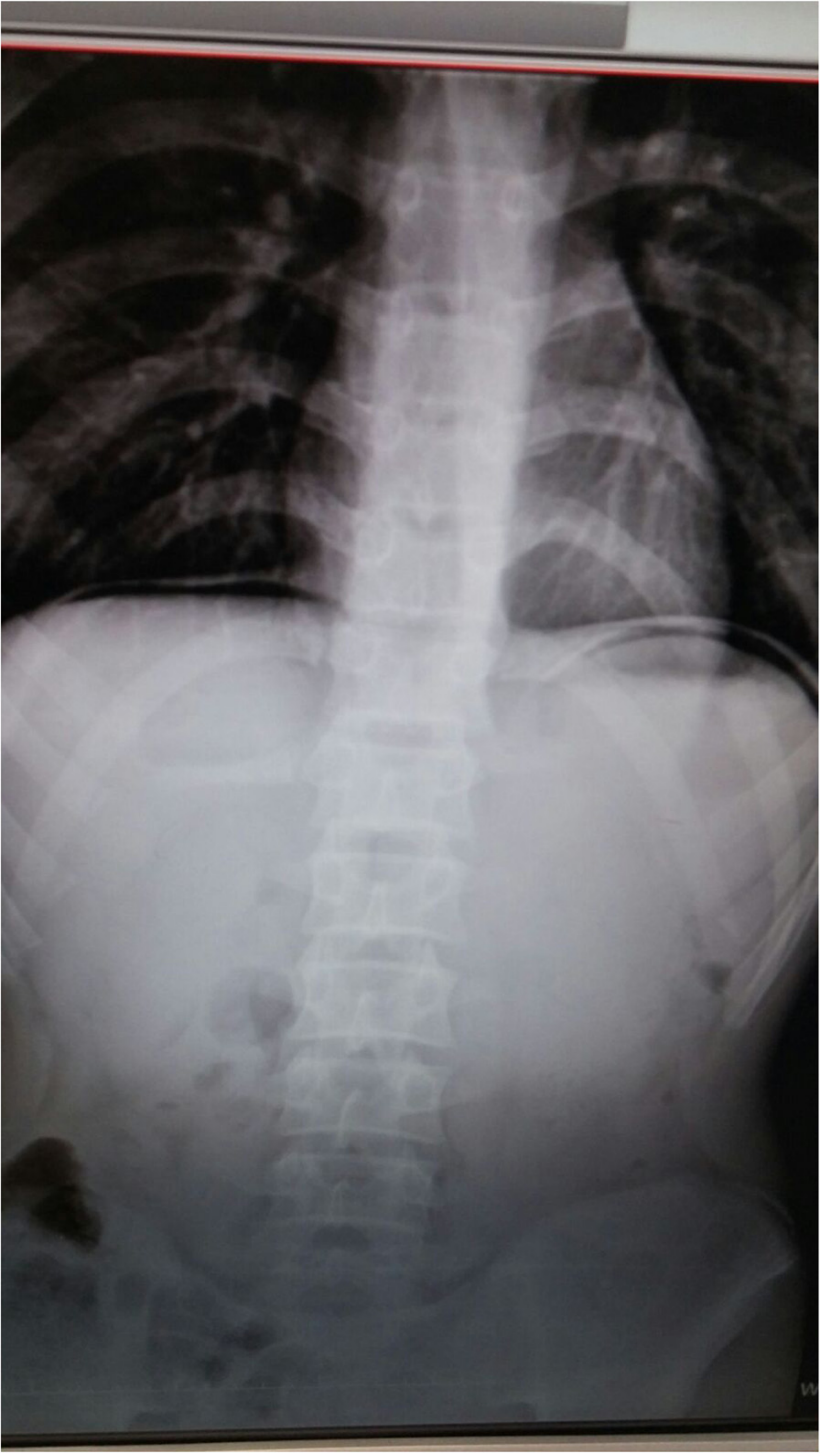
Plain abdominal radiogram: subdiaphragmatic free air

**Fig. 2:**
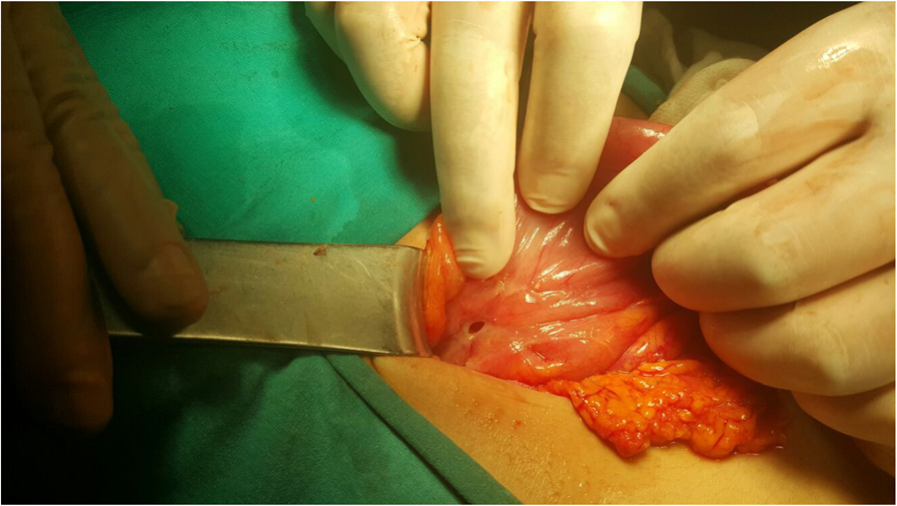
Perforated area of the first part of the duodenum

Plain abdominal radiogram in standing position demonstrated subdiaphragmatic free air (Fig. 1). In nasogastric decompression, bilious drainage was observed. In abdominal ultrasound (US), free fluid collections were detected between bowel loops and also between the liver and the duodenum. Exploration through midline incision revealed the presence of diffuse pus and free fluid in the abdominal cavity. All bowel loops were covered with fibrin, and a perforated area on the first part of the duodenum measuring nearly 1 cm in diameter was detected (Fig. 2). Primary closure was performed using an omental patch (Graham patch), and abdominal cavity was irrigated with physiologic saline. A drain was placed in the subhepatic region, and the abdomen was closed in compliance with proper surgical principles. Postoperative course progressed without any complication. Antibiotherapy and gastroprotective medication were used. On the 5th postoperative day, oral alimentation was started, and his drain was removed on the 7th postoperative day. He was discharged with the prescription of oral antibiotherapy and proton pump inhibitors.

### Discussion

Since PUD is a rarely seen entity in adolescents, most of the physicians do not suspect the presence of this clinical diagnosis in a child who is referred to the emergency service with complaint of abdominal pain. Acid and pepsin are important factors in the development of PUD. Stress, trauma, major surgeries, *Helicobacter pylori* infection, burns, familial predisposition, systemic diseases, and some drugs (NSAIDs and steroids) are other predisposing factors [5]. However, in our case, none of these predisposing factors were found. From the patient’s history, it was learnt that he was a bonsai user for 2 years. Perforation is one of the serious complications detected in bonsai users, and diagnosis is usually made following the development of a complication.

Pathognomonic findings of this condition which was firstly defined by Travers in the year 1817 are sudden onset of abdominal pain, generalized peritonitis, and washboard abdomen which were also present in our case. In nearly 90 % of the patients, subdiaphragmatic free air is found, as is the case with our patient [6]. In the US, we also detected intraabdominal free fluid.

In addition to their some therapeutic effects, SCs can demonstrate many deleterious effects. More than ten kinds of SCs have been produced so far, and bonsai is just one of them [3]. As is known, it is widely used among youngsters. According to the World Drug Report released from the United Nations Office on Drugs and Crime, SCs have been the most prevalently used new psychoactive substances worldwide [7]. SCs were imported in Turkey in the year 2010, and their use is gradually increasing [3].

By binding to cannabinoid binding (CB) receptors, SCs imitate the effects of natural cannabinoids. CB1 and CB2 receptors are mainly found in neurons and immune-mediated cells; however, their presence in GIS has been also confirmed. In animal studies, inhibitory effects of cannabinoids and CB1 receptor agonists on gastric motility and contractility have been demonstrated. In clinical use, they exert their therapeutic effects via CBI receptors in the treatment of hyperemesis in patients receiving chemotherapy. Paradoxically, Choung et al. [8] emphasized the presence of a correlation between their chronic use and hyperemesis.

Besides, cannabinoids bind to CBI receptors in *nucleus tractus solitarius* with potential resultant effects on lower esophageal sphincter. Their diverse effects on the stomach have been also demonstrated. They also delay gastric emptying. In the literature, a patient who developed acute gastric dilation and hepatic portal venous gas was also reported [9]. Administration of a cannabinoid receptor antagonist anandamide to mice with cerulein-induced pancreatitis increased the severity of pancreatitis, a phenomenon which cannot be explained clearly.

Effect of cannabinoids on the pancreatic canal and Oddi’s sphincter may contribute to aggravation of pancreatitis [10]. The effect of cannabinoids on gastric secretions and emptying could be the cause of gastric and duodenal ulcers present in our patient. We think that cannabinoids binding to CB1 receptors led to the development of this condition. As is seen in our case, in the literature, duodenal perforations due to formation of ulcerations generally had small sizes (0.5–1 cm), and they could be treated easily. In this case report, we presented an unusual manifestation of chronic SC use.

In our case, Naranjo score was +3; the mean of this score is possible adverse drug reactions [11].

We achieved this score from the answers of the questions shown below.

1. Are there previous conclusive reports on this reaction? = yes (+1 point).
2. Did the adverse reaction improve when the drug was discontinued? = yes (+1 point).
3. Are there alternative causes that could have caused the reaction? = no (+1 point).

The other questions answers were as follows: do not know or not done (0 point).

## Conclusions

Development of duodenal perforation due to the chronic use of SC is an unusual condition. These patients can consult to the emergency service with recurrent abdominal pain. Since their use is forbidden by law, the patients do not tell that they are using SC in their anamnesis. In order to determine etiology, it is important to investigate the use of addictive substances which are increasingly popular among adolescent cases admitted to the emergency services with PUD or its complications.

## Consent

Written informed consent was obtained from the patient’s legal guardian(s) for the publication of this case report and accompanying images. A copy of the written consent is available for review by the Editor-in-Chief of this journal.
